# Robotic Rectosigmoid Resection with Totally Intracorporeal Colorectal Anastomosis (TICA) for Recurrent Ovarian Cancer: A Case Series and Description of the Technique

**DOI:** 10.3390/jpm14101052

**Published:** 2024-10-11

**Authors:** Valerio Gallotta, Luca Palmieri, Francesco Santullo, Camilla Certelli, Claudio Lodoli, Carlo Abatini, Miriam Attalla El Halabieh, Marco D’Indinosante, Alex Federico, Andrea Rosati, Carmine Conte, Riccardo Oliva, Anna Fagotti, Giovanni Scambia

**Affiliations:** 1 Gynecologic Oncology Unit, Department of Woman and Child Health and Public Health, Fondazione Policlinico Universitario A. Gemelli IRCCS, 00168 Rome, Italy; valerio.gallotta@policlinicogemelli.it (V.G.);; 2 Gynecologic Oncology Unit, Department of Woman and Child Health and Public Health, Fondazione Policlinico Universitario A. Gemelli IRCCS, Università Cattolica del Sacro Cuore, 00168 Roma, Italy; luca.palmieri02@icatt.it (L.P.);; 3 Surgical Unit of Peritoneum and Retroperitoneum, Fondazione Policlinico Universitario A. Gemelli IRCCS, L.go A. Gemelli, 00168 Rome, Italy

**Keywords:** minimally invasive surgery, rectum recurrence, intracorporeal colorectal anastomosis, ovarian cancer, robotic surgery

## Abstract

Background: Most patients with ovarian cancer relapse within 2 years. Prospective randomized trials, such as DESKTOP III and SOC-I, have shown the role of secondary cytoreduction in improving oncological outcomes in selected patients, when complete tumor resection is achieved. Recent retrospective series suggest that minimally invasive surgery is a feasible option in oligometastatic recurrences, such as rectal ones. Methods: Five patients with an isolated rectal recurrence infiltrating the bowel wall underwent a robotic rectosigmoid resection with totally intracorporeal colorectal anastomosis. The procedure began with retroperitoneal access to manage the vascular structures, followed by visceral resection with a minimally invasive approach. The standard steps of an en-bloc pelvic resection, including intracorporeal end-to-end anastomosis, were performed. The treatment data were evaluated. Results: The mean age of the patients was 54 years, and their mean body mass index was 30. All patients had at least one previous abdominal surgery and 60% had high-grade serous ovarian cancer at their initial diagnosis. Their mean platinum-free interval was 17.4 months. Complete secondary cytoreduction was achieved in all cases, with histopathology confirming bowel infiltration. The mean procedure duration was 294 min, with an estimated blood loss of 180 mL. No intraoperative complications occurred. The mean hospital stay was 8 days. One patient had a grade 2 postoperative complication. The mean follow-up period was 14 months, with only one patient experiencing a recurrence at the level of the abdominal wall. Conclusions: Robotic rectosigmoid resection is a viable option for complete cytoreduction in isolated recurrent ovarian cancer.

## 1. Introduction

Despite the advances in cytoreductive efforts and the potential addition of bevacizumab and PARP inhibitors to front-line platinum-based regimens, the vast majority of patients with ovarian cancer relapse and succumb to the disease within five years of their initial diagnosis [1,2,3,4,5,6]. The standard treatment of recurrent ovarian cancer (ROC) primarily relies on medical therapy, chosen based on “platinum sensitivity”; this crucial parameter, traditionally defined by the interval between the completion of first-line chemotherapy and disease relapse, has been recently recognized as having a greater level of complexity. It is in fact influenced by histological type, the status of BRCA genes or Homologous Recombination Deficiency (HRD), previous antiangiogenetic treatment, the pattern of the relapse’s presentation, and other factors, thus suggesting potential as-yet-unknown scenarios for future treatments [7,8,9,10]. Indeed, a treatment plan is selected on the basis of multiple factors including the anatomical site of the relapse, infiltrative pattern, primary treatment(s), chemo- and/or radio sensitivity, and the clinical characteristics of patients [11,12,13,14,15]. Several retrospective studies, as well as a recent prospective randomized trial, have suggested that secondary cytoreductive surgery could provide better clinical outcomes in platinum-sensitive ROC patients, in cases of complete tumor cytoreduction, which is essential for delivering a true clinical benefit [16,17,18,19,20,21,22,23,24,25,26]. Consequently, it is challenging to select which patients with recurrent disease will benefit from surgery; indeed, the role of surgery in the recurrent setting is completely different compared to upfront treatment, thus requiring a careful balance between expected benefits and potential morbidity [17,18,19,20,21,22,23,24,25,26,27,28,29,30,31,32].

About 50% of patients with recurrent ovarian cancer have a pelvic component to their disease and 22% of patients present an isolated pelvic recurrence, often involving the rectum. Some biological and clinical evidence suggests that isolated rectum recurrences from ovarian cancer would be better managed with surgery than medical treatment [33,34]. There is evidence that minimally invasive secondary cytoreductive surgery is associated with favorable perioperative outcomes, with no differences seen in terms of post-recurrence survival compared to an open approach in recurrent ovarian cancer [35,36,37,38,39,40].

Robotic technology provides considerable advantages in terms of stable 3D vision, 360° movements, tremor filtering, the ergonomic position of the surgeon, and intraoperative ultrasound and indocyanine green application [37,41]. Based on this hypothesis, integrating a robotic approach into minimally invasive secondary cytoreduction could markedly improve the management of isolated recurrent ovarian cancer and related clinical outcomes [42,43,44].

Moreover, the synergy within a multidisciplinary team of different physicians such as radiologists, pathologists, anesthesiologists, and surgeons can lead to improved patient selection for minimally invasive personalized surgery [45].

The main objectives of this retrospective study are to assess the feasibility of a robotic surgical approach in recurrent ovarian cancer, to accurately describe the surgical technique of totally intracorporeal colorectal anastomosis, and to evaluate the oncological outcomes.

## 2. Material and Methods

In this single-center retrospective study, we enrolled five patients with recurrent ovarian cancer who underwent secondary cytoreductive surgery with a robotic modified en-bloc rectosigmoid resection.

All patients underwent surgery at Fondazione Policlinico Universitario A. Gemelli Istituti di Ricovero e Cura a Carattere Scientifico (IRCCS), Department of Gynaecologic Oncology, from January 2022 to January 2024.

The study was approved by the local Ethics Committee (ID 6663/Protocol 0010182/24).

All patients with ovarian cancer recurrence underwent evaluation by a multidisciplinary tumor board, which is composed of gynecologic oncologic surgeons, oncologists, radiologists, pathologists, and anesthesiologists, to assess the most appropriate treatment.

**Figure 1:**
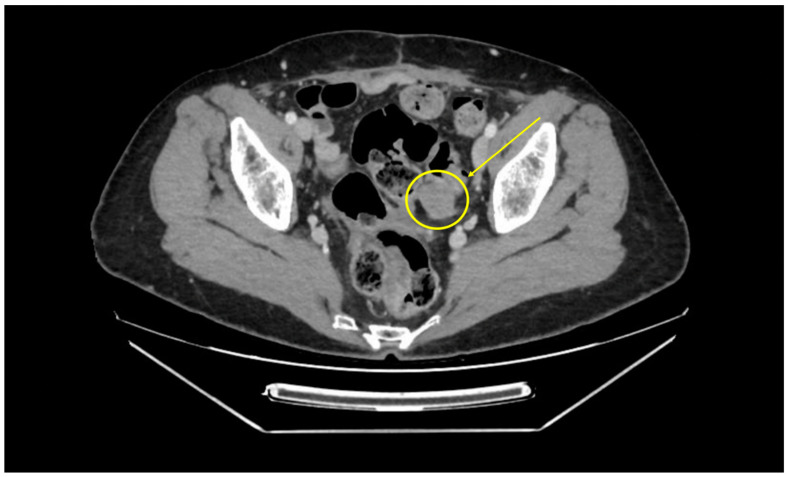
Pre-operative CT-SCAN, analyzed during preoperative multidisciplinary board, showing a rectal lesion of about 3–4 cm adherent to the left parametrium, ureter, and internal iliac vessels at the level of the vaginal stump.

Once a surgical treatment was chosen, the parameters influencing the decision to use a surgical approach included age, clinical conditions (patient’s ability to tolerate the Trendelenburg position and pneumoperitoneum), medical history (previous surgeries or radiotherapies), body mass index (BMI), and the pattern of recurrence (Figure 1).

The inclusion criteria for robotic secondary cytoreduction were as follows: platinum-sensitive disease (platinum-free interval (PFI) ≥ 6 months), resectable pelvic disease evaluated by computed tomography (CT) scan and/or positron emission tomography (PET), an American Society of Anesthesiologists classification score ≤ 2 (ASA ≤ 2), and the provision of informed written consent. The exclusion criteria were as follows: extra-abdominal metastasis, the presence of diffuse carcinomatosis, and an ASA ≥ 2.

All the procedures were performed with the Da Vinci Xi System (Intuitive Surgical, Sunnyvale, CA, USA).

Data about the patients’ medical history, including their age, body mass index, number of previous surgeries, neo-adjuvant chemotherapy, type of first surgery, residual disease, histology, International Federation of Gynecology and Obstetrics (FIGO) stage, BRCA status, Homologous Recombination Deficiency status, number of recurrences, and platinum-free interval, were collected.

Perioperative outcomes including operative time, estimated blood loss during surgery, intraoperative complications, and hospital stay duration, as well as adjuvant therapy, were evaluated. The estimate of the blood lost was based on a visual assessment, observing the graduated suction canister. Postoperative complications were investigated up to 30 days post-surgery and they were classified according to Clavien-Dindo classification [46].

All patients underwent follow-up examinations according to ESGO-ESMO-ESP recommendations, which included a physical examination and CA-125 determination every three months and CT scan evaluation every six months for the first two years. From the third to the fifth year after surgery, the follow-up included a physical examination, CA-125 determination, and CT scan evaluation every six months. Subsequently, the same methods were used once a year.

### Operative Technique

The treatment plan consisted of a robotic rectosigmoid resection with total intracorporeal colorectal anastomosis (TICA).

**Figure 2:**
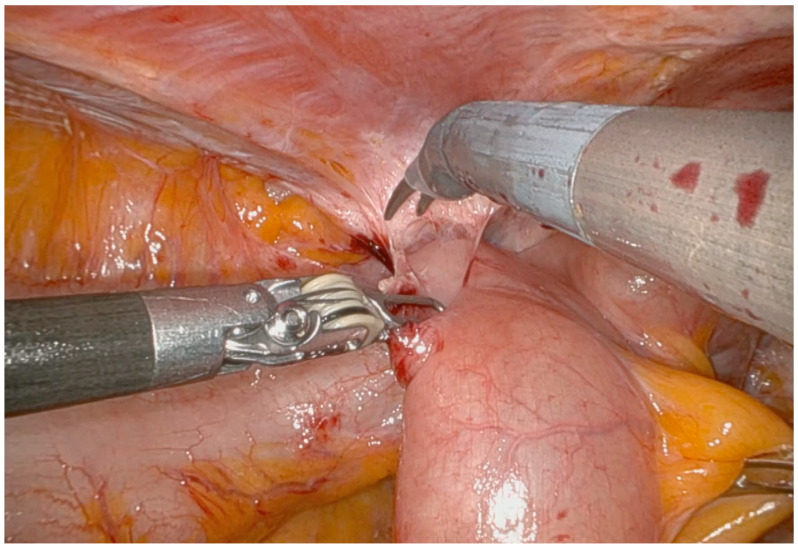
Adhesiolysis procedures; small bowel loops are attached to the abdominal wall.

Surgery begins with careful adhesiolysis, which is a crucial step in restoring anatomy altered by previous surgeries. This also allows for the placement of robotic trocars when necessary and confirms the absence of carcinomatosis. Robotic surgery enhances adhesiolysis procedures, as its detailed and precise visualization of the anatomy can reduce intraoperative complications, particularly bowel injuries (Figure 2) [37,41].

**Figure 3:**
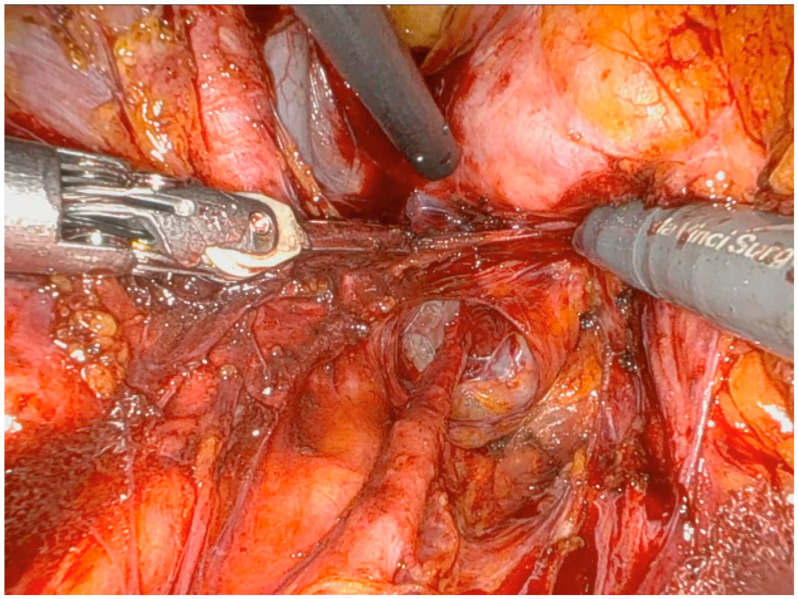
Retroperitoneal space; we can identify the ureter, which is fully isolated, and the iliac vessels.

The first surgical step is to reach the retroperitoneal space. Retroperitoneal fibrosis, related to previous surgeries, is managed with the careful manipulation of pelvic structures. The sigmoid colon and rectum are mobilized from the vascular planes by developing pararectal and paravesical spaces and performing accurate ureter dissection (Figure 3).

**Figure 4:**
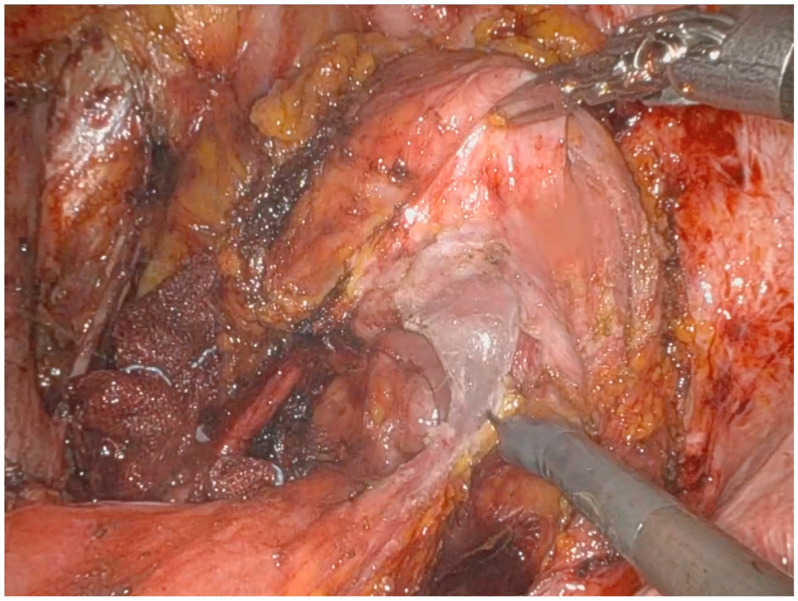
Recto-vaginal septum preparation; ventrally, the bladder and the posterior vaginal wall are observable, while the rectum is visible posteriorly.

Afterwards, it is crucial to expose the posterior vaginal wall by developing a rectovaginal space, allowing for the identification of the inferior limit of the dissection and complete mobilization of the rectum (Figure 4).

**Figure 5:**
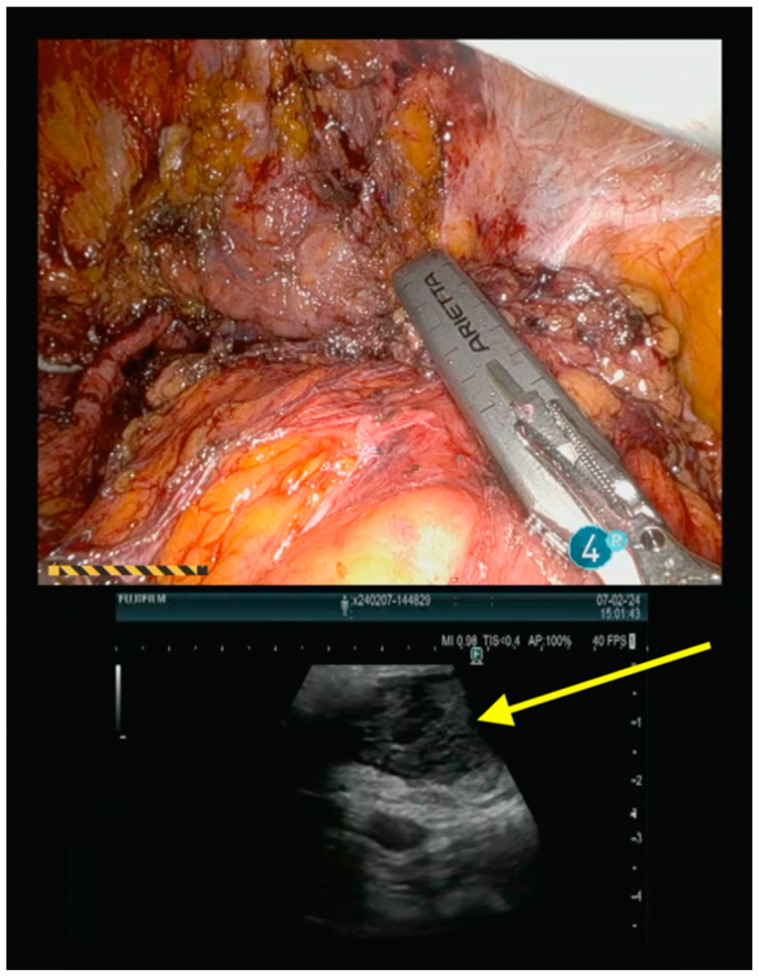
Intraoperative ultrasound, showing a 4 cm vascularized lesion of the sigma rectum. The precise localization of the recurrence allows for complete disease resection with clear surgical margins.

Robotic intraoperative ultrasound is useful for the real-time intraoperative evaluation of the boundaries of the lesion to ensure complete disease resection (Figure 5).

The sigmoid colon and rectum are dissected 4–5 cm away from tumor margins in order to achieve a rectosigmoid resection with clear surgical margins.

**Figure 6:**
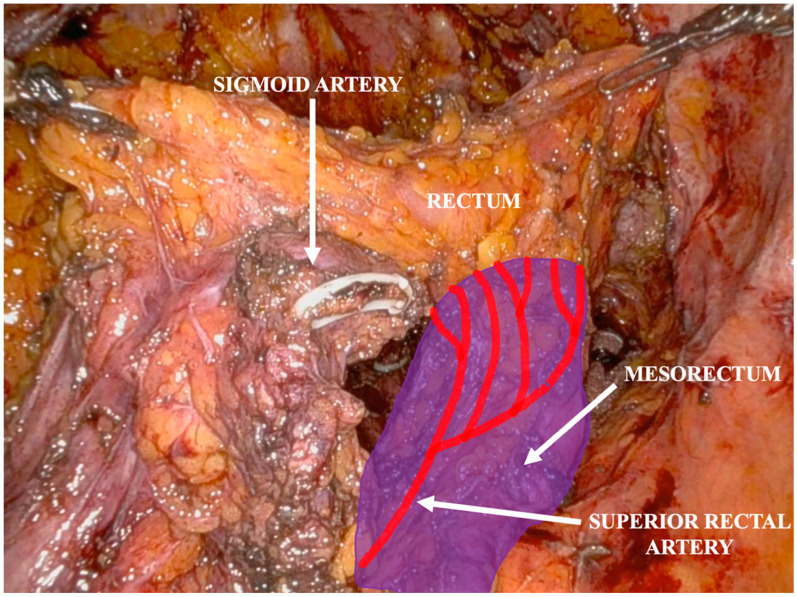
Sigmoid vessels are selectively ligated with an endoclip, and the mesorectum is fully preserved.

The sigmoid arteries are ligated with an endoclip near the bowel wall, preserving the inferior mesenteric artery (IMA) (Figure 6).

**Figure 7:**
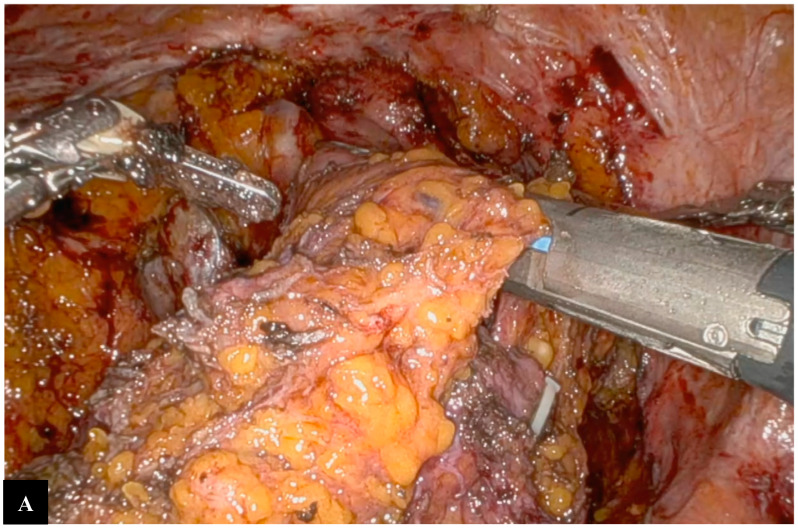

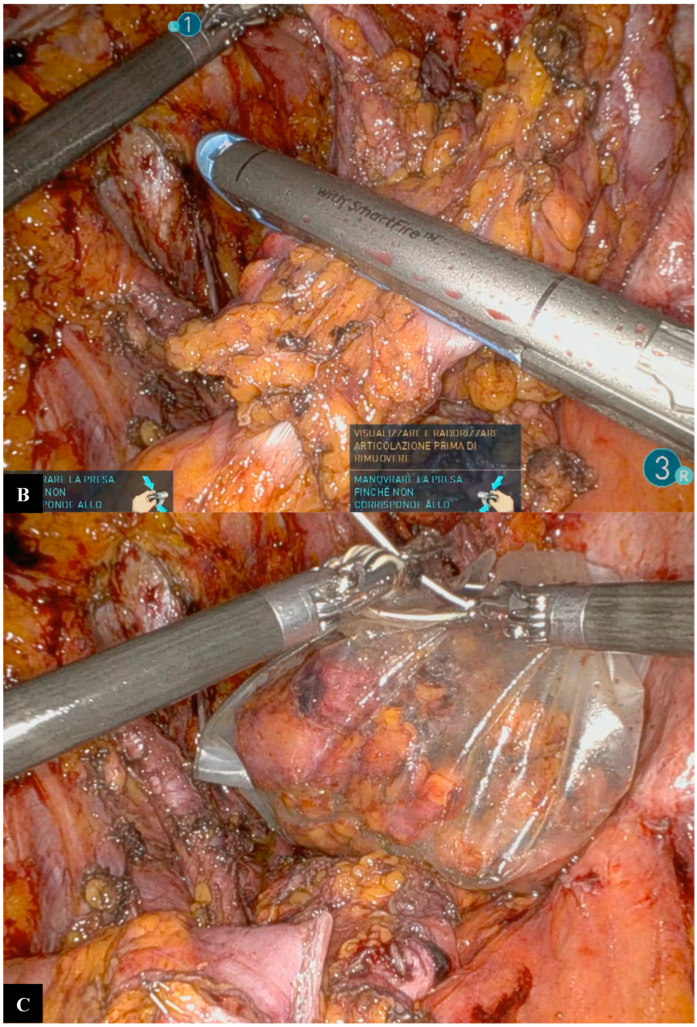
Bowel resection. (**A**) Resection downstream from the tumor, (**B**) Resection upstream from the tumor, (**C**) Portion of rectosigmoid colon removed in endo-bag.

A mesorectal-sparing rectosigmoid resection is then performed using a robotic linear-powered stapler. Finally, the rectum is divided, and the surgical specimen is removed using an endo-bag (Figure 7).

**Figure 8:**
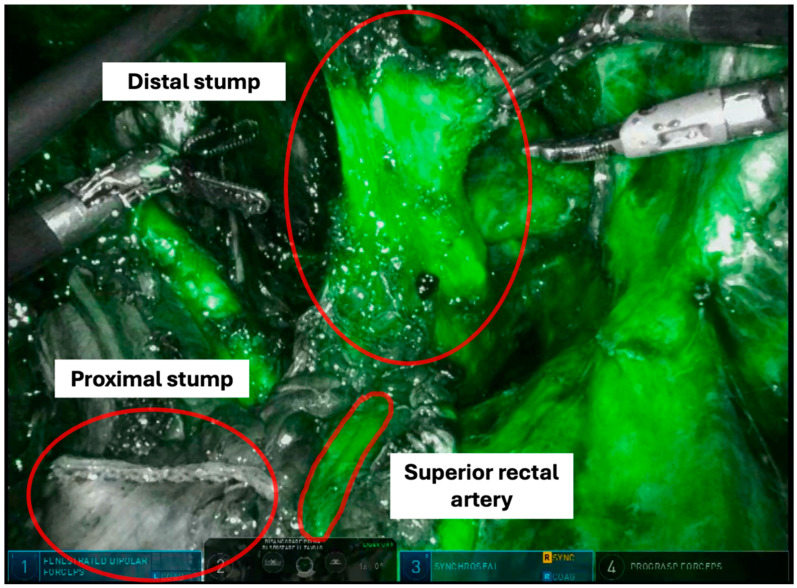
Indocyanine green fluorescence angiography at the site of resection.

The vascularization of the bowel stumps is checked through intravenous indocyanine green injection. This also allows for the evaluation of the preservation of the superior rectal artery, a branch of the inferior mesenteric artery (Figure 8).

**Figure 9:**
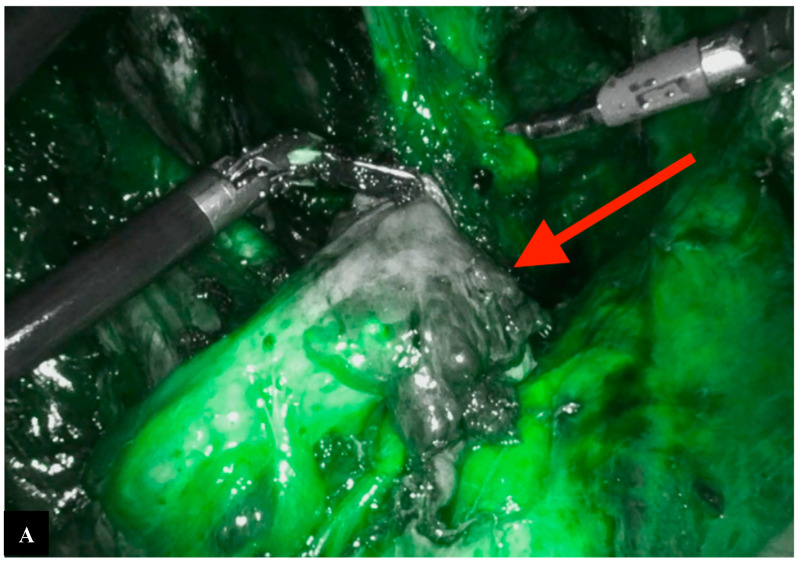

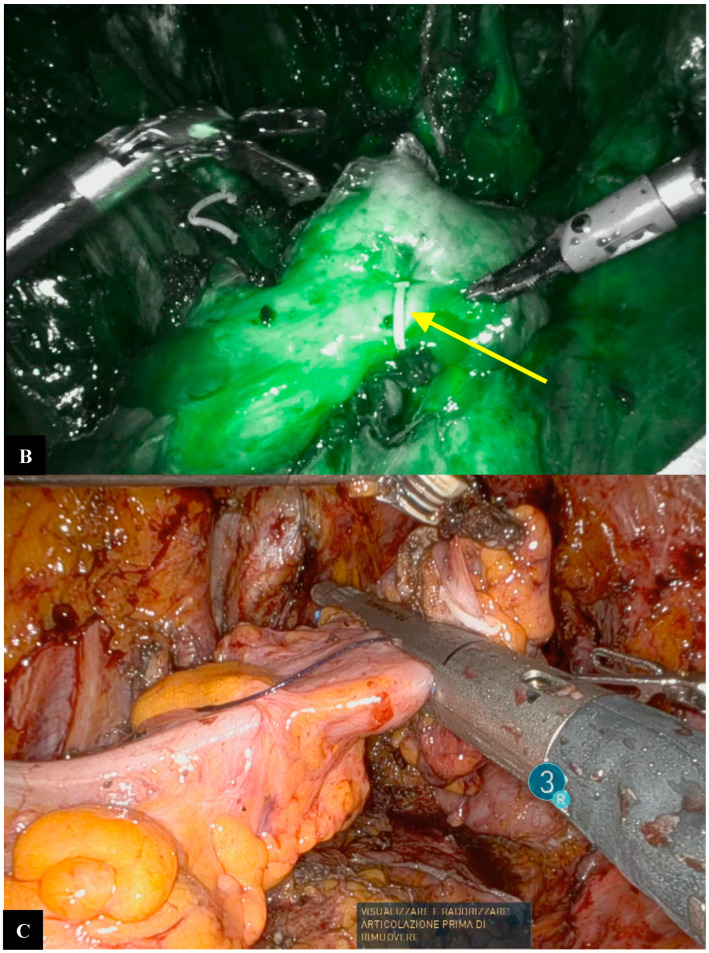
Resection of the portion of the descending colon with inadequate perfusion. (**A**) Hypoperfused area of the proximal syump, (**B**) An endoclip is applied to mark the boundary of the hypoperfused area, (**C**) Resection upstream from the endoclip.

If a hypoperfused area is identified by an absence of indocyanine green, it will be resected to ensure a well-vascularized colorectal anastomosis (Figure 9).

**Figure 10:**
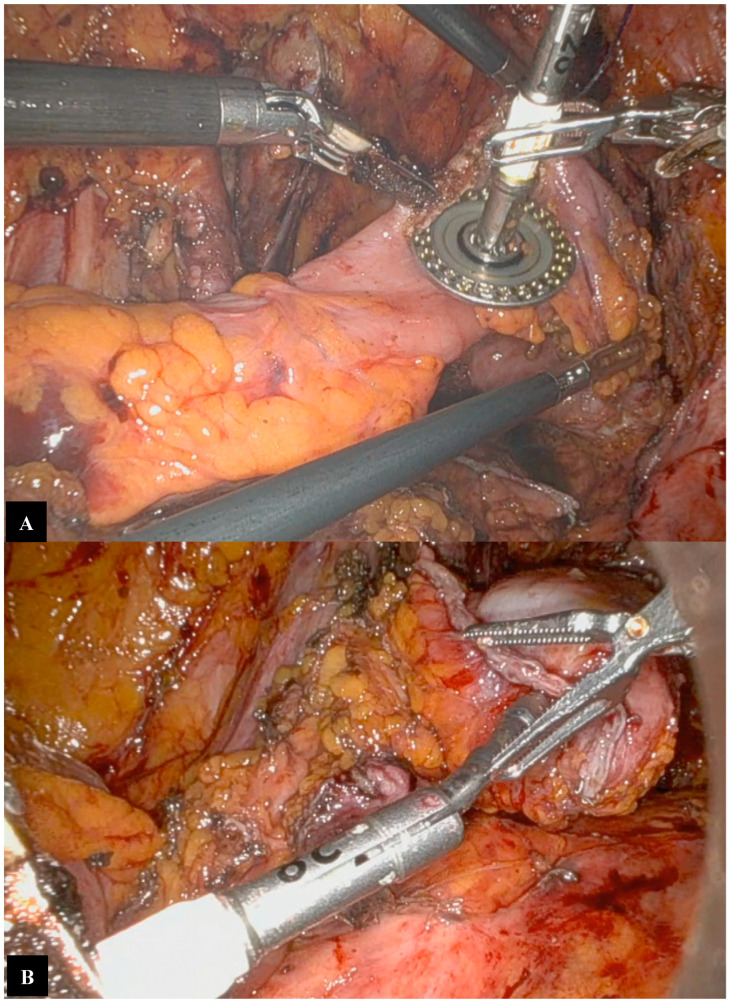

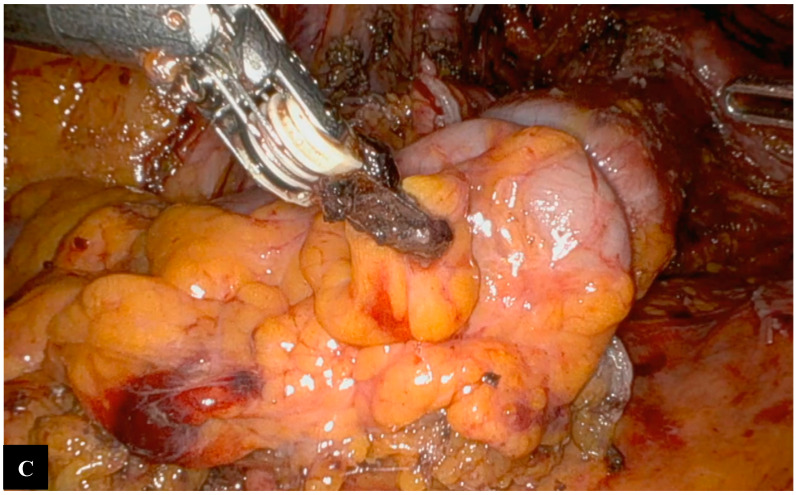
Totally intracorporeal colorectal anastomosis (TICA). (**A**) The anvil of the stapler is introduced in the abdomen through the subcostal trocar and inserted into the proximal stump. (**B**) The circular powered stapler is passed through the anus and coupled with the anvil on the descending colon. (**C**) Total intracorporeal colorectal anastomosis (TICA).

The anvil of the stapler is introduced into the abdomen through the assistant trocar and inserted into the proximal stump through a small incision in the bowel wall. The circular stapler is passed through the anus and coupled with the anvil on the descending colon and a totally intracorporeal colorectal anastomosis (TICA) is performed (Figure 10).

**Figure 11:**
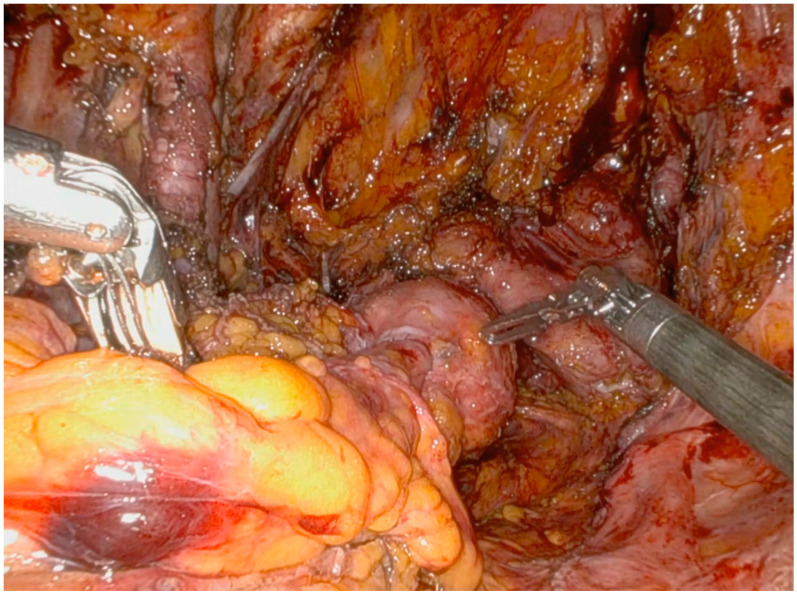
Intraoperative bobwel leak test with povidone-iodine.

To evaluate the stapled colorectal anastomosis, the proximal and distal tissue rings are examined for completeness. Finally, an intraoperative leak test is performed. This involves the instillation of air or fluid (e.g., methylene blue, saline, or povidone-iodine) per rectum while maintaining intraluminal pressure by occluding the bowel proximal to the anastomosis (Figure 11).

**Figure 12:**
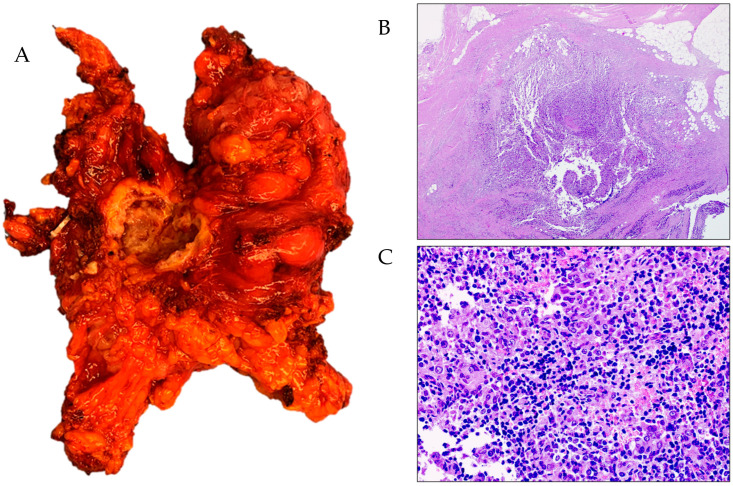
Surgical specimen and final histologic examination. (**A**) Surgical specimen including segment of sigmoid colon and rectum, 18 cm in length, site of whitish nodular area 3 cm in diameter at the level of the bowel serosa. (**B**,**C**) Intestinal tissues with ab-extrinsic infiltration by carcinoma with morphological features consistent with high-grade serous histotype.

The specimen is then removed through an enlargement of the assistant trocar and sent for final histologic examination (Figure 12).

## 3. Results

Five patients were included in this study. Their patient characteristics are reported in Table 1. The mean age of the patients was 54 years (range: 46–65 years). The mean body mass index (BMI) was 30 (range: 21–30). All the patients had a history of at least one previous abdominal surgery. One patient had a chronic kidney disease and had undergone kidney transplantation twice. All of the primary surgeries had left no residual tumor, and in none of them was a rectosigmoid resection performed. In three cases, a Douglas peritonectomy was reported. High-grade serous ovarian cancer (HGSOC) was the predominant histological type and it was detected in three (60%) cases. All the patients underwent platinum-based adjuvant chemotherapy. In case number three, a maintenance therapy with niraparib was administered. After a mean platinum-free interval (PFI; the time between the last platinum chemotherapy and recurrence) of 17.4 months (range 10–28 months), the five patients experienced a rectal recurrence. The mean diameter of their lesions was 26 mm (range 15–50).

Perioperative outcomes are shown in detail in Table 2. The mean duration of the procedure and estimated blood loss were 294 min (range: 210–480 min) and 180 mL (range 100–300 mL), respectively. No intraoperative complications occurred. No protective ileostomies were performed. The mean hospital stay was 8 days (range: 6–13 days). None of the patients received blood transfusions. Only one patient experienced a grade 2 postoperative complication: abdominal collection and a urinary tract infection treated by intravenous antibiotics.

In all cases, complete cytoreduction was achieved and the histopathologic results confirmed the extrinsic invasion of a carcinoma in the bowel wall. Additionally, in every case, the resection margins were free of cancer, confirming complete disease resection.

Two patients received platinum-based chemotherapy, two patients received letrozole, and one patient continued maintenance therapy with niraparib. The mean follow-up time after the secondary cytoreductions was 14 months (range: 5–28 months). One patient experienced a recurrence of disease at the level of the abdominal wall during the time of observation and was treated by further surgery. This case involved a non-epithelial ovarian tumor, specifically a granulosa cell tumor. All patients were alive and with no evidence of disease at the time of this manuscript’s submission.

## 4. Discussion

The present study shows, for the first, time that robotic rectosigmoid resection with a totally intracorporeal colorectal anastomosis is a feasible surgical procedure for achieving complete cytoreduction in recurrent ovarian cancer.

To achieve complete cytoreduction consistent with the requirements of the cancer no-touch isolation technique, we proposed a modified en-bloc pelvic resection and totally intracorporeal bowel reconstruction. This modified resection involves initially accessing the retroperitoneal space for central vascular ligation and mobilizing the rectum from surrounding pelvic structures. The accurate identification of anatomical structures and their boundaries, as well as the careful preparation of narrow pelvic structures, is crucial in minimally invasive rectosigmoid resections.

The no-touch isolation technique was adopted to minimize the risk of tumor spillage and prevent the dissemination of tumor cells, as described in previous studies on other solid abdominal tumors [47,48]. Theoretically, the no-touch isolation technique aims to reduce the spread of cancer cells from the primary tumor site to the peritoneal cavity and other organs. This is achieved by first ligating the blood and lymphatic vessels and avoiding tumor manipulation [41,42,43,44,45,46,47,48,49,50,51]. The proposed retroperitoneal approach should, if not decrease intraoperative cancer’s dissemination, at least reduce complications such as blood loss and intraoperative issues, while allowing for complete vascular control. The surgery concludes with a TICA, which offers two main advantages in robotic secondary cytoreduction. First, there is no need for extensive mobilization of the colon to bring it out through the Pfannenstiel incision, which decrease the risk for mesenteric bleeding and serosal injuries, thereby preserving a significant portion of the descending colon and reducing the risk of complications, such as anastomotic leakage. Second, by avoiding an open approach, there is less scarring, better clinical outcomes, and a faster recovery, as reported in our experience [52,53,54,55,56,57,58,59,60,61,62].

This surgical technique, applied to a highly selected group of patients, is subject to important considerations due to its unique clinical setting, making comparisons difficult. We derived this approach from our initial experience with minimally invasive rectal resection for deep endometriosis [63,64,65]. However, patients with a rectal recurrence of ovarian cancer present particular challenges, as it is often not an isolated lesion and may present carcinomatosis. We have adapted this surgical technique, used as primary approach in rectal cancer, to the unique pattern of the dissemination and recurrence of ovarian cancer, which often involves managing a bulky disease and extensive adhesions.

Regarding the robotic approach, this technology allows for the reproduction of the same surgical steps as traditional surgery, with the benefits of a minimally invasive technique, overcoming the limitations of a laparoscopy: an unstable video camera, limited range of instrument movements, two-dimensional imaging, and poor ergonomics for the surgeon [66].

Moreover, the robotic system facilitates the identification of anatomical structures and simplifies some complex surgical steps in narrow spaces such as the pelvis, allowing for the integrated use of surgical tools such as intraoperative ultrasound and indocyanine green applications [67,68]. Conversion to a laparotomy was never required in our series, despite the presence of adhesions in all patients, and no intraoperative complications were observed.

Some limitations of our study must be acknowledged.

Firstly, the main limitations are represented by its retrospective nature, relatively short follow-up time, and the small number of patients enrolled. Despite the promising results, further studies are needed to evaluate the robotic approach as a standard surgical strategy for the treatment of isolated colorectal recurrences in ROC patients.

In the end, what can we take from this initial experience of robotic secondary rectum resection? A few things should be recognized, including that minimally invasive secondary cytoreduction in very selected patients could be considered feasible and safe, although only in select centers and by select surgeons proficient in such an approach.

Moreover, in our opinion, the early detection of pelvic recurrence and an appropriate multidisciplinary preoperative assessment, including a molecular characterization of the disease, radiological analysis of preoperative images, and anesthesiologic evaluation, are crucial for improving outcomes in patients with recurrent ovarian cancer.

## 5. Conclusions

In conclusion, a robotic rectosigmoid resection is a feasible and safe option for isolated bowel recurrences, but accurate patient selection is essential.

In the coming years, deep learning and artificial intelligence could significantly enhance the role of secondary cytoreductive surgery, especially through preoperative 3D reconstructions and the integration of clinical, pathological, and molecular data.

Gynecologic oncologists will need to expand their expertise in challenging resections, especially in complex surgeries for ROC patients, and will need to intensify their translational research and testing of a new approach tailored to improving the fight against solid tumors. The era of “precision” surgery for ROC has arrived.

## Figures and Tables

**Table 1:** Patients’ characteristics.

	Age	BMI	Number of Pervious Surgeries	NACT	FIRST Surgery	Hystology	FIGO Stage	BRAC Status	Number of Recurrencec	PFI
**1**	49	40	1	no	LPT: TH + BSO + omentectomy + pelvic and aortic LND + appendectomy + WP	HGSOC	IIIA1(ii)	WT	I	19
**2**	46	35	1	no	LPT BSO Restaging LPT: LND + omentectomy	Granulosa cell tumor	IIIB	-	II	10
**3**	56	24	2	yes	IDS-LPT: TH + BSO + omentectomy + Douglas peritonectomy	HGSOC	IIIC	WT	I	28
**4**	64	24	4	yes	IDS-LPT: TH + BSO + omentectomy + Douglas peritonectomy + splenctomy + HIPEC	HGSOC	IVB	WT	I	11
**5**	57	26	1	no	LPT: TH + BSO + omentectomy + appendectomy + Douglas peritonectomy	Endometrioid G1	IIA	-	I	19

BMI: body mass index, BSO: bilateral salpingo-oophorectomy, HGSOC: high-grade serous ovarian cancer, HIPEC: hyperthermic intraperitoneal chemotherapy, LND: lymphadenectomy, LPT; laparotomy, NACT: neoadjuvant chemotherapy, PFI: platinum-free interval, RT: residual tumor, TH: total hysterectomy, WP: peritoneal washing, WT: wild type.

**Table 2:** Perioperative outcomes.

	Operative Time (min)	EBL (mL)	Intraoperative Complications	Hospital Stay (Days)	Adjuvant Therapy	FUP (Months)	Status
**1**	480	200	no	6	VI cycles Carboplatin-Caelyx + Rucaparib	28	alive
**2**	300	300	No	9	Letrozole	16	Recurrence at 6 months; alive
**3**	210	200	no	6	VI cycles Carboplatin	16	alive
**4**	270	100	no	7	Niraparib continuation	7	alive
**5**	210	100	no	13	Letrozole	5	alive

EBL: estimated blood loss, FUP: follow-up.

## Data Availability

Data are contained within the article.
